# Mitochondrial Complex I Function Is Essential for Neural Stem/Progenitor Cells Proliferation and Differentiation

**DOI:** 10.3389/fnins.2019.00664

**Published:** 2019-06-26

**Authors:** Daniel Cabello-Rivera, Helia Sarmiento-Soto, José López-Barneo, Ana M. Muñoz-Cabello

**Affiliations:** ^1^Instituto de Biomedicina de Sevilla (IBiS), Hospital Universitario Virgen del Rocío, CSIC, Universidad de Sevilla, Seville, Spain; ^2^Facultad de Medicina, Departamento de Fisiología Médica y Biofísica, Universidad de Sevilla, Seville, Spain; ^3^Centro de Investigación Biomédica en Red sobre Enfermedades Neurodegenerativas (CIBERNED), Madrid, Spain

**Keywords:** mitochondrial dysfunction, neurogenesis, neural stem cell, metabolism, oxidative phosphorylation

## Abstract

Neurogenesis in developing and adult mammalian brain is a tightly regulated process that relies on neural stem cell (NSC) activity. There is increasing evidence that mitochondrial metabolism affects NSC homeostasis and differentiation but the precise role of mitochondrial function in the neurogenic process requires further investigation. Here, we have analyzed how mitochondrial complex I (MCI) dysfunction affects NSC viability, proliferation and differentiation, as well as survival of the neural progeny. We have generated a conditional knockout model (hGFAP-NDUFS2 mice) in which expression of the NDUFS2 protein, essential for MCI function, is suppressed in cells expressing the Cre recombinase under the human glial fibrillary acidic protein promoter, active in mouse radial glial cells (RGCs) and in neural stem cells (NSCs) that reside in adult neurogenic niches. In this model we observed that survival of central NSC population does not appear to be severely affected by MCI dysfunction. However, perinatal brain development was markedly inhibited and *Ndufs2* knockout mice died before the tenth postnatal day. In addition, *in vitro* studies of subventricular zone NSCs showed that active neural progenitors require a functional MCI to produce ATP and to proliferate. *In vitro* differentiation of neural precursors into neurons and oligodendrocytes was also profoundly affected. These data indicate the need of a correct MCI function and oxidative phosphorylation for glia-like NSC proliferation, differentiation and subsequent oligodendrocyte or neuronal maturation.

## Introduction

Generation of neurons (neurogenesis) and glial cells (gliogenesis) in the developing central nervous system (CNS) are tightly regulated processes that rely on the activity of neural stem and progenitor cells as well as on extracellular signals provided by the surrounding niche (Taverna et al., 2014; Bond et al., 2015). In mammals, neuroepithelial cells from the neural tube are the primary NSCs that in early development give rise to RGCs, which are responsible for the generation of mature neurons and glia in the CNS (Kriegstein and Álvarez-Buylla, 2009). Neurogenesis, delimited to some central and peripheral areas, can also take place in adulthood (Ming and Song, 2005; Pardal et al., 2007; Kriegstein and Álvarez-Buylla, 2009; Dimou and Götz, 2014). The extent of adult neurogenesis and its physiological relevance vary among the different mammalian species (Amrein et al., 2011; Frisén, 2016; Kempermann et al., 2018). Embryonic and adult NSCs self-renewal, proliferation and differentiation are regulated by both intrinsic and extrinsic mechanisms at multiple levels, implicating a wide variety of signaling cascades and modulated through specific transcriptional programs and epigenetic cues (Bond et al., 2015; Khacho et al., 2019). The role of mitochondrial metabolism and dynamics in embryonic and adult neurogenesis has gained considerable attention in recent years (Ito and Suda, 2014; Feng and Liu, 2017; Knobloch and Jessberger, 2017). Indeed, metabolic plasticity seems to play a pivotal role in the regulation of NSCs vital processes (Folmes et al., 2012; Folmes and Terzic, 2014; Khacho and Slack, 2018). It has been extensively accepted that NSCs are mostly glycolytic, whereas a metabolic switch from glycolysis to mitochondrial oxidative phosphorylation (OxPhos) is essential for neuronal differentiation and survival (Homem et al., 2014; Khacho et al., 2016; Zheng et al., 2016). However, there is increasing evidence supporting the hypothesis that mitochondrial function is not only essential for post-mitotic neuron survival, but also for other aspects of neural stem and progenitor cells homeostasis, affecting survival and proliferation of these cell populations (Díaz-Castro et al., 2015; Beckervordersandforth et al., 2017; Khacho et al., 2017; Khacho and Slack, 2018).

Despite significant progress has been made toward understanding how mitochondrial metabolism regulates the neurogenic process, the precise requirement of mitochondrial OxPhos in neural stem and progenitor cells requires further investigation. In this study, we examined the effect of genetically induced MCI dysfunction on NSCs and their progeny. We generated a mouse model with conditional deletion of the *Ndufs2* gene, which encodes a mitochondrial subunit that contributes to the ubiquinone/rotenone binding site and is necessary for the assembly and catalytic activity of MCI (Fernández-Agüera et al., 2015; Arias-Mayenco et al., 2018). To target the neurogenic cell populations with the Cre/lox system, we used the human glial fibrillary acidic protein (hGFAP) promoter which is active both in the murine RGCs (Malatesta et al., 2000, 2003) and in the adult NSCs that reside in the subventricular zone (SVZ) and the subgranular zone of the dentate gyrus in the hippocampus (Pastrana et al., 2009; Beckervordersandforth et al., 2010, 2014). Using this MCI dysfunction model (hGFAP-NDUFS2 mice) we observed that brain development was markedly affected whereas the peripheral nervous system did not seem to be altered. In addition, *in vitro* analysis of perinatal neural stem and progenitor cells showed that a correct MCI function is needed for glia-like neural stem and progenitor cell proliferation, differentiation and subsequent oligodendrocyte or neuronal maturation.

## Materials and Methods

### Animal Models

Mice were housed at regulated temperature (22 ± 1°C) with *ad libitum* access to drink and food in a 12/12 h light/dark cycle. The animals were maintained before, during and after the experiments according to EUROPEAN DIRECTIVE 2010/63/EU regarding the use of experimental animals and other scientific purposes (ROYAL DECREE 53/2013, February 8). All procedures were reviewed and approved by the Ethics Committee of Animal Experimentation (CEEA/CEI) of Hospital Virgen del Rocío/Institute of Biomedicine of Seville (reference number 22-09-15-332). hGFAP-NDUFS2 knockout mice (*Ndufs2^flox/-^* hGFAP-Cre genotype) were generated by breeding the *Ndufs2*-flox strain (Fernández-Agüera et al., 2015) with the transgenic strain hGFAP-Cre (Zhuo et al., 2001). Experimental mice where F1 hybrids from a C57BL/6 × 129/SV cross. P0 and P7 male and female mice were used in this study. Genotypes were confirmed by PCR analysis as described previously (Díaz-Castro et al., 2015; Fernández-Agüera et al., 2015). For simplicity, the results from the hGFAP-NDUFS2 mice littermates (*Ndufs2^flox/+^, Ndufs2^flox/-^, Ndufs2^flox/+^* hGFAP-Cre) were pooled together where indicated and assigned to a control group, as no differences were detected among them. hGFAP-tdTomato mice were obtained by breeding hGFAP strain with the Ai14 mice (Madisen et al., 2010). For the neurosphere assays in the presence of rotenone, P30 wild type animals in the C57BL/6 background were used. For euthanasia, mice were anesthetized by intraperitoneal injection of sodium thiopental at a lethal dose of 120–150 mg/kg of animal weight.

### Tissue Preparation and Histological Analysis

Dissected brains were fixed overnight in 4% paraformaldehyde (PFA) prepared in phosphate-buffered saline (PBS) and embedded in paraffin. Coronal brain sections (20 μm thick) were obtained with the aid of a microtome (Leica) and were used for NeuN, ki67, cleaved caspase-3 or GFAP immunostaining. Immunohistochemical detection was performed using the EnVision + System-HPR (Dako) following the manufacturer instructions. Sections were incubated overnight at 4°C with either of the following antibodies: rabbit anti-GFAP antibody (Dako; 1:200), rabbit anti-cleaved caspase-3 (Cell Signaling; 1:100), rabbit anti-Ki67 (Thermo Scientific; 1:200) and mouse anti-NeuN (Millipore; 1:500). For GFAP quantitative analysis, immunofluorescence detection was performed using a rabbit anti-GFAP (Dako; 1:100) as the primary antibody, and Alexa Fluor 568 goat-anti-rabbit IgG as a secondary antibody. Nuclei were detected by 0.5 μg/mL 4′,6′-diamidino-2-phenylindole (DAPI) counterstaining. Sections were mounted on Leica CV Mount and visualized using the Olympus BX61 microscope (Olympus). Dissected carotid bifurcations and adrenal glands were fixed in 4% PFA for 3 h. Tissues were embedded in OCT (Tissue-Tek) after sucrose (30% w/v in PBS) cryoprotection and sectioned (10 μm thick) with a cryostat (Leica). Peripheral tissue sections were used for tyrosine hydroxylase (TH) immunodetection as previously described (Platero-Luengo et al., 2014; Díaz-Castro et al., 2015). A rabbit anti-TH (Novus; 1:1000) was used as primary antibody. For fluorescence detection, Alexa Fluor 568 donkey-anti-rabbit IgG or Alexa Fluor 488 donkey-anti-rabbit IgG antibodies were used. Nuclei were detected by 0.5 μg/mL DAPI counterstaining. Sections were mounted on Fluorescence mounting medium (Dako) and visualized using an Olympus BX61 microscope. Confocal images were acquired with a Leica SP2-AOBS confocal Microscope. ImageJ software (National Institutes of Health) was used for blinded cell counting and stained area quantification. The Cavalieri principle was applied for volume estimation.

### SVZ Neurosphere Assay

The neurosphere assays were performed as previously described (d’Anglemont de Tassigny et al., 2015). Briefly, the SVZ area was isolated from the walls of the lateral ventricles in freshly dissected mouse brains and submerged in ice-cold PBS. The pieces of the tissue were incubated for 20 min at 37°C in a 5% CO_2_, 20% O_2_ humidified atmosphere in papain solution: 22 U/mL papain (Sigma), 0.5 mM EDTA, 1 mM L-cysteine and 0.5 mg/mL DNase I (Sigma) in Earle’s balanced salt solution (EBSS) (GIBCO). After digestion, SVZ slices were mechanically dissociated with fire-polished Pasteur pipette in trypsin inhibitor solution containing 2.5 mg/mL trypsin inhibitor (Sigma), 25 mg/mL bovine serum albumin (Sigma), 20 mM glucose and 23 mM NaHCO_3_ in EBSS to quench papain. Cells were centrifuged for 5 min at 300 × *g*, washed and resuspended in neurosphere culture medium (NCM): Dulbecco’s Modified Eagle’s Medium-F12 (GIBCO) containing 100 U/mL penicillin/streptomycin, 1% (v/v) N2 and 2% (v/v) B27 supplements (GIBCO), 10 ng/mL basic fibroblast growth factor (R&D Systems), 20 ng/mL epidermal growth factor (R&D Systems) and 0.7 U/mL heparin (Sigma). Dispersed cells were counted and plated in ultralow-attachment 6-well plates (Corning Inc.) at a 2.5 cells/μL clonal density, to obtain free-floating cultures of primary neurospheres. Cells were placed in the 5% CO_2_, 20% O_2_, 37°C incubator for 7 days. When indicated, neurosphere cultures where supplemented with 10 nM rotenone (Sigma), 2 mM sodium pyruvate (GIBCO), 0.1 mg/mL uridine and/or dimethyl succinate (2 or 5 mM) (Sigma). The number of floating colonies per well was counted without *a priori* information on the genotype and the percentage of neurosphere-forming cells was calculated. Images from each well and condition were acquired on an inverted IX71 Olympus microscope and the diameter of the neurospheres was calculated using ImageJ software. For differentiation assays, mitogens from NCM were removed and neurospheres were plated under adherent conditions in glass fibronectin (7 μg/mL) treated-coverslips. After 2, 5, and 7 days under differentiation conditions, cells were fixed in 4% PFA for 15 min at room temperature. Inmunofluorescence detection was done as previously described (d’Anglemont de Tassigny et al., 2015). Antibodies and the dilution factors used were as follows: mouse anti-TUJ1 (Millipore; 1:1000), rabbit anti-GFAP (Dako; 1:500) and rabbit anti-NG2 (Millipore; 1:200). For fluorescence detection, Alexa Fluor 568 donkey-anti-mouse IgG and Alexa Fluor 488 donkey-anti-rabbit IgG were used. Nuclei were detected by 0.5 μg/mL DAPI counterstaining. Coverslips were mounted on Fluorescence mounting medium (Dako) and visualized using the Olympus BX61 microscope. Cells were counted blinded. Areas were selected based on DAPI staining and then TUJ1, NG2, and GFAP expression was analyzed.

### RNA Extraction and Quantitative Real-Time PCR

Dissected tissues were fast-frozen with liquid N_2_ and stored at -80°C. Total RNAs were isolated with either Trizol reagent (Life technologies) for brain samples or RNeasy Micro Kit (Qiagen) for carotid body (CB), superior cervical ganglion (SCG), and SVZ neurospheres following the manufacturer instructions. Each CB and SCG replicate was obtained from pooled samples from 3 to 4 mice for each genotype. In addition, CB RNA was amplified using GeneChip^TM^ WT Pico Kit (Thermo Fisher Scientific). Reverse transcription of RNA was performed using the QuantiTect Reverse Transcription Kit (Qiagen). Real-time quantitative PCR reactions were performed in a 7500 Fast Real Time PCR System (Thermo Fisher Scientific) using a TaqMan Gene Expression Assay (Thermo Fisher Scientific) for each specific gene. For *Ndufs2* expression analysis a TaqMan Gene Expression Assay for exon 2–3 boundary was used. Glyceraldehyde 3-phosphate dehydrogenase (*Gapdh*) gene was analyzed to normalize the samples.

### Mitochondrial Complex I Activity

Mitochondrial Complex I activity in the dorsal cortex of the brain was estimated using the Complex I enzyme activity dipstick assay kit (Abcam). Dorsal cortex was isolated from freshly dissected coronal brain slices and stored frozen at -80°C until processing. Tissue lysates were obtained by mechanical homogenization (Dounce homogenizer) in 200 μL of the extraction buffer supplemented with protease inhibitor and phosphatase inhibitor cocktails (Sigma). The homogenates were incubated on ice for 20 min and then centrifuged at 16000 × *g* for 30 min. The supernatant was collected for the enzymatic assay and protein concentration was determined by Bradford assay (BioRad). To measure MCI activity, 5 μg of each protein extract was used. Images from the developed dipsticks were acquired (ImageQuant LAS 4000 mini, GE Healthcare) and signal intensity was quantified using the ImageQuant TL software (GE Healthcare). Interpolation from a standard curve was performed.

### ATP Measurement

One week-cultured neurospheres were collected and washed in PBS. After 5 min centrifugation at 300 × *g*, neurospheres were resuspended in homogenization solution (100 mM Tris, 4 mM EDTA, pH 7.75). Cells were disrupted with a homogenizer (Omni 2000; Omni International) and boiled for 3 min followed by centrifugation (1 min at 1000 × *g*). Supernatant was collected for ATP measurement and samples were diluted and normalized using the absorbance at 280 nm. ATP levels were determined using ATP Bioluminescence Assay CLS II kit (Roche Applied Science) following the manufacturer instructions.

### Microfluorimetric Recordings

One week-cultured neurospheres were seeded on glass fibronectin-treated coverslips and incubated for at least 2 h in NCM. Coverslips with neurospheres were transferred to a recording chamber in an inverted microscope (Nikon eclipse Ti) equipped with a 40×/0.60 NA objective and a filter wheel, a 150 W xenon lamp, a monochromator, a CCD camera and a computer. Neurospheres were continuously perfused with external solution on the recording chamber. Experiments were performed at 30–33°C. The bathing solution was composed of (in mM): 125 NaCl, 23 NaHCO_3_, 5 Glucose, 5 Sucrose, 4.5 KCl, 2.5 CaCl_2_, and 1 MgCl_2_. The external solution was bubbled with a gas mixture of 5% CO_2_, 20% O_2_, and 75% N_2_. A monochromator (Polychrome V, Till Photonics) and a dichroic mirror (FF409-Di03, Semrock) were used as the excitation system. The emitted fluorescence was detected with a CCD camera (Orca Flash 4, Hamamatsu Photonics) after passing through a band-pass filter (FF01-510/84, Semrock). A non-ratiometric protocol was applied to measure NAD(P)H autofluorescence (Muñoz-Cabello et al., 2018). NAD(P)H has an excitation peak at 360 nm and emits at 460 nm. The acquisition protocol was designed with a spatial resolution of 4 × 4 pixels, an excitation time of 150 ms (λ = 360 nm), and an acquisition interval of 5 s. Background fluorescence was subtracted in all the experiments. The value of the emitted fluorescence by each selected cell at the beginning of the experiment was considered as the basal NAD(P)H level. As a control for the specificity of the NAD(P)H detection, at the end of each recording, cells were exposed to 1 mM alpha-ketobutyrate (αKB), a metabolite that induces a rapid decrease in NADH levels (see Arias-Mayenco et al., 2018 for details). Monochromator, CCD camera and image acquisition were controlled by AQUACOSMOS software (Hamamatsu Photonics). The analysis of the data was done with Igor Pro program.

### Statistical Analyses

Data are presented as mean ± SEM. The observed distribution of genotypes was compared to the expected Mendelian ratios by Chi-square test. Unpaired Student’s *t* test analysis was used when indicated. The One Way analysis of variance (ANOVA) or the Two Way ANOVA was used followed by the Tukey’s *post hoc* test to compare multiple samples. Multiple *t*-test analysis was used for two-group comparisons. Differences with the level of significance *p* < 0.05 were considered significant. Statistical analyses were performed using Prism 6.0 (GraphPad Software).

## Results

### Genetic Inactivation of Mitochondrial Complex I in Neural Stem Cells Results in Brain Developmental Alterations and Perinatal Death

**FIGURE 1:**
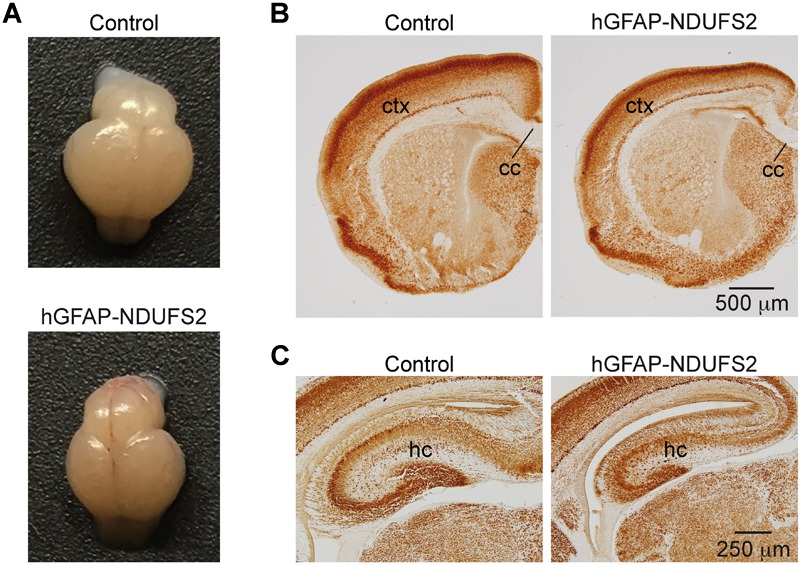
Brain analysis of control and hGFAP-NDUFS2 P0 mice. **(A)** Photographs of *Ndufs2^flox/+^* control (top) and hGFAP-NDUFS2 (bottom) P0 mice brains. **(B,C)** Coronal brain sections immunostained with NeuN from representative *Ndufs2^flox/+^* control (left panels) and hGFAP-NDUFS2 (right panels) P0 mice. cc, corpus callosum; ctx, cortex; hc, hippocampus.

**FIGURE 2:**
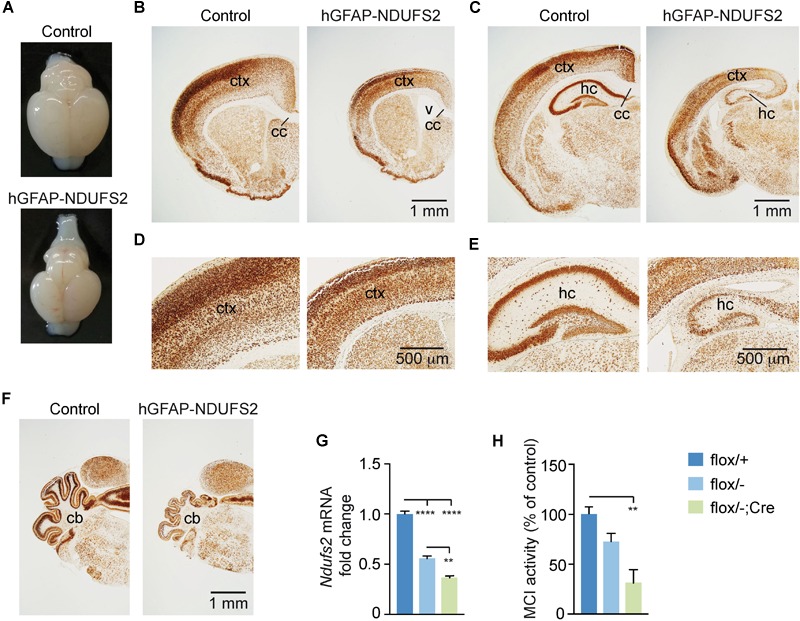
Brain analysis of control and hGFAP-NDUFS2 P7 mice. **(A)** Photographs of *Ndufs2^flox/+^* control (top) and hGFAP-NDUFS2 (bottom) P7 mice brains. **(B–F)** Coronal brain sections of representative P7 *Ndufs2^flox/+^* control (left panels) and hGFAP-NDUFS2 (right panels) mice immunostained for NeuN neuronal marker. Images in panels **(D,E)** are higher magnifications of panels **(B,C)**, respectively. **(G)** Relative *Ndufs2* mRNA levels in dorsal telencephalon of P7 mice (*n* = 3 mice/group). **(H)** MCI activity in dorsal telencephalon of P7 mice (*n* = 3–4 mice/group). Data are presented as mean ± SEM. ^∗∗^*p* < 0.01, ^∗∗∗∗^*p* < 0.0001 (One Way ANOVA and Tukey’s *post hoc* test). cc, corpus callosum; cb, cerebellum; ctx, cortex; hc, hippocampus; v, ventricle.

**FIGURE 3:**
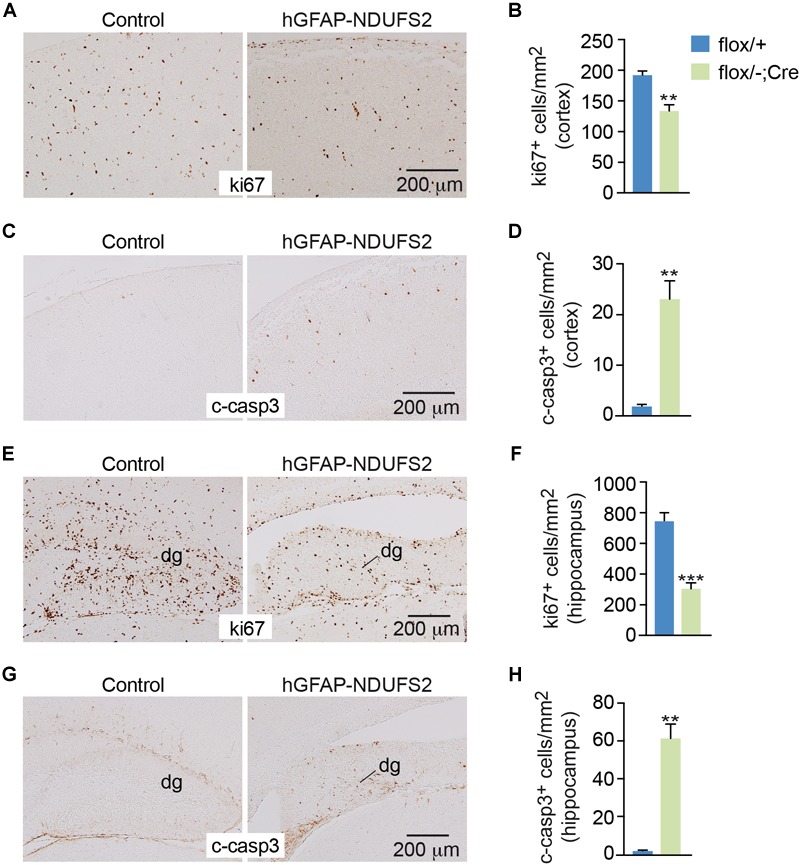
Reduced cell proliferation and increased apoptosis in the brain of hGFAP-NDUFS2 mice. **(A)** Coronal brain sections showing ki67 immunostaining in the cortex of *Ndufs2^flox/+^* control (left) and hGFAP-NDUFS2 (right) P7 mice. **(B)** Quantitative analysis of ki67^+^ cortical cells from P7 mice (*n* = 3 mice/group). **(C)** Cleaved caspase-3 immunodetection in the cortex of *Ndufs2^flox/+^* control (left) and hGFAP-NDUFS2 (right) P7 mice. **(D)** Quantitative analysis of cleaved-caspase-3^+^ cortical cells from P7 mice (*n* = 3 mice/group). **(E–H)** Representative images of coronal brain sections immunostained for ki67 **(E)** or cleaved-caspase-3 **(G)** in the hippocampus of *Ndufs2^flox/+^* control (left) and hGFAP-NDUFS2 (right) P7 mice and quantitative analysis of ki67^+^ **(F)** or cleaved-caspase-3^+^ **(H)** cells in the hippocampus from P7 mice (*n* = 3 mice/group). Data are presented as mean ± SEM. ^∗∗^*p* < 0.01, ^∗∗∗^*p* < 0.001 (unpaired Student’s *t*-test analysis). c-casp3, cleaved caspase-3; dg, dentate gyrus.

**FIGURE 4:**
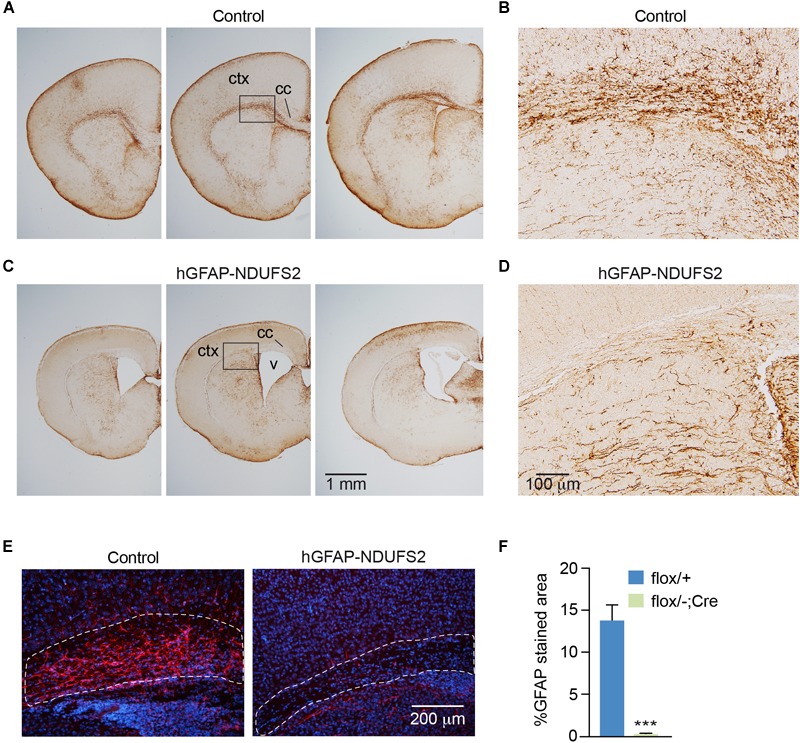
GFAP immunodetection in control and hGFAP-NDUFS2 brains. **(A–D)** Coronal brain sections of P7 *Ndufs2^flox/+^* control **(A,B)** and hGFAP-NDUFS2 **(C,D)** mice immunostained with a GFAP antibody. Images in panels **(B)** and **(D)** are higher magnifications of the areas selected in panels **(A)** and **(C)**, respectively. **(E)** Immunofluorescence detection of GFAP (red) in corpus callosum of *Ndufs2^flox/+^* (control, left) and hGFAP-NDUFS2 (right) coronal brain sections from P7 mice. **(F)** Quantitative analysis expressed as percentage of GFAP^+^ stained area relative to the selected areas (dotted lines in the images from panel **E**). Nuclei were counterstained with DAPI (blue). Data are presented as mean ± SEM (*n* = 3 mice/group). ^∗∗∗^*p* < 0.001 (unpaired Student’s *t*-test analysis). cc: corpus callosum; ctx, cortex; v, ventricle.

To evaluate the impact of MCI dysfunction on neurogenesis we used animals carrying *Ndufs2* flox/– alleles and the hGFAP-Cre transgene (hGFAP-NDUFS2 mice). Mutant mice were born at the expected Mendelian ratio (Supplementary Figure S1A) and appeared healthy at the moment of birth (Supplementary Figure S1B). Although at postnatal day (P) 0 hGFAP-NDUFS2 mice were apparently indistinguishable from littermates and their brains were macroscopically similar to controls (Figure 1A), the histological analyses revealed a decrease in cortical thickness (Figure 1B) and subtle hippocampal abnormalities (Figure 1C). At around P5, hGFAP-NDUFS2 mice experimented a rapid worsening, showing decreased body size (Supplementary Figure S1C) and onset of ataxia, and died between P7 and P9. At this stage, we observed a marked reduction in the brain size of hGFAP-NDUFS2 mice, in which recombination efficiency was assessed (Figure 2). hGFAP-NDUFS2 brains showed profound anatomical abnormalities that were more evident in dorsal cortical areas, the hippocampus and cerebellum (Figure 2B–F). *Ndufs2* knockout mice frequently displayed ventricle dilatation and corpus callosum atrophy (Figure 2B). These results are in agreement with previous lineage tracing studies based on hGFAP-Cre mediated recombination (Malatesta et al., 2003; Anthony and Heintz, 2008) that resulted in the labeling of radial glial-derived neurons in the dorsal, but not the ventral, telencephalon (see section “Discussion”). *Ndufs2* mRNA levels in dorsal telencephalon decreased in heterozygous (*Ndufs2^flox^*^/^*^-^*) and in hGFAP-NDUFS2 mice to ∼55 and ∼35% respecting the values seen in the homozygous (*Ndufs2^flox/+^*) controls (Figure 2G). Accordingly, MCI activity was markedly reduced in cells of the dorsal telencephalon from hGFAP-NDUFS2 mice (Figure 2H). Atrophy of the affected brain regions could be caused by a decline of proliferation and/or an increase in cell death. In order to clarify the involvement of those processes in the hGFAP-NDUFS2 brain phenotype, staining of the ki67 marker was used to identify proliferating cells and apoptosis was evaluated by activated caspase-3 immunodetection (Figure 3). hGFAP-NDUFS2 mice showed a decreased number of ki67^+^ cortical cells (Figure 3A,B) and exhibited a remarkable induction of cortical apoptosis (Figure 3C,D). Reduced proliferation and increased cell death were also clearly seen in the hippocampus of the knockout mice (Figure 3E–H). These results revealed that neonatal neurogenesis is disrupted in hGFAP-NDUFS2 mice because both proliferation and programmed cell death processes are altered. Interestingly, immunohistological analyses using a specific GFAP antibody revealed that GFAP^+^ cells were present in the hGFAP-NDUFS2 brains (Figure 4). However, marked differences were seen in the *Ndufs2* knockout mice regarding the morphology and distribution of these cells. GFAP^+^ cells with long processes, that could represent a more undifferentiated population, were typically present in the brain (particularly in the striatum) of knockout mice (Figure 4A–D). On the other hand, the population of fibrous astrocytes in the corpus callosum was profoundly affected (Figure 4B,D–F). Together, these data indicate that gliogenesis and neurogenesis were deeply affected in hGFAP-NDUFS2 mice, suggesting an important role of mitochondrial OxPhos in these processes.

### Effect of NDUFS2-Deficiency on Peripheral Neural Tissues

**FIGURE 5:**
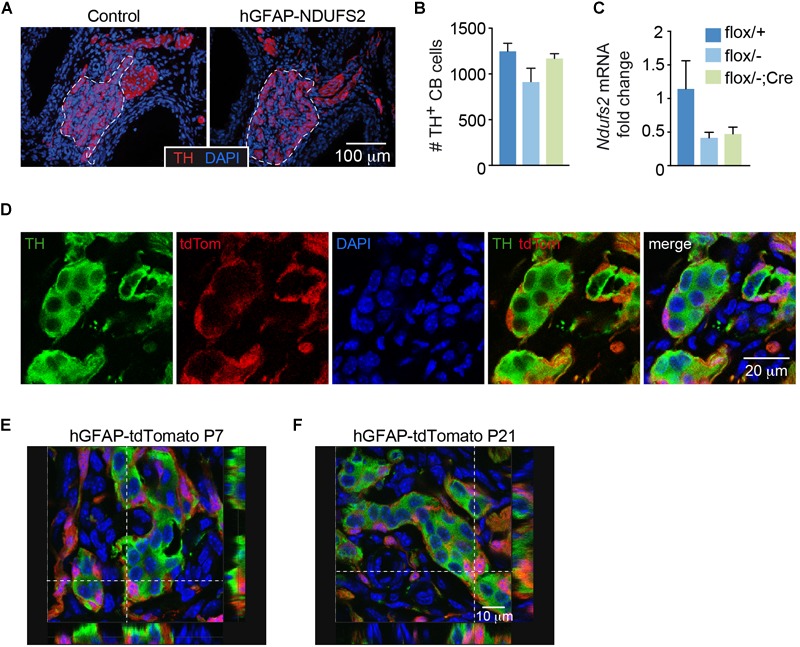
Carotid body analysis in control and hGFAP-NDUFS2 mice. **(A)** Immunofluorescence detection of TH^+^ glomus cells (red) in CB sections from *Ndufs2^flox/+^* control (left) and hGFAP-NDUFS2 (right) P7 mice. Nuclei were counterstained with DAPI (blue). CB parenchyma is delimited by dotted lines. **(B,C)** Number of TH^+^ cells **(B)** (*n* = 3–9 mice/group) and *Ndufs2* mRNA levels **(C)** (*n* = 3 independent experiments from pooled samples) in CBs from P7 mice. Data are presented as mean ± SEM (One Way ANOVA and Tukey’s *post hoc* test). **(D)** Confocal images detecting tdTomato (red) and TH (green) in CB sections from a representative P7 hGFAP-tdTomato mouse. Nuclei were counterstained with DAPI (blue). **(E,F)** Ortogonal projections of confocal images detecting TH (green), tdTomato (red) and DAPI (blue) in CB sections from P7 **(E)** and P21 **(F)** hGFAP-tdTomato mice.

In parallel with the studies on brain development, we studied whether neurogenesis and/or neuronal viability in the peripheral nervous system were affected in hGFAP-NDUFS2 mice. We did not detect any significant morphological abnormalities in neural crest-derived catecholaminergic autonomic organs such as the SCG (Supplementary Figures S2A,B) or the adrenal medulla (AM) (Supplementary Figures S2D,E). The lack of hGFAP-Cre dependent activity in SCG was confirmed by quantitative PCR analysis of *Ndufs2* mRNA levels (Supplementary Figure S2C), supporting previous observations indicating that SCG sympathetic neurons and AM chromaffin cells do not derive from GFAP^+^ cells (Díaz-Castro et al., 2015). We also searched for potential alterations in the carotid bodies (CBs), paired neural crest-derived and O_2_-sensitive dopaminergic organs that undergo final maturation during the 3–4 postnatal weeks (Carroll and Kim, 2013). Adult CBs can grow during sustained hypoxia due to a resident population of glia-like multipotent stem cells (Pardal et al., 2007), hence it seemed to us relevant to examine whether developmental postnatal CB expansion of the neuron-like glomus cell population depends on the GFAP^+^ progenitors and if MCI dysfunction alters their postnatal generation and/or survival. Similar to SCG or AM, we did not appreciate any change in CB morphology (Figure 5A) or in the number of TH positive glomus cells (Figure 5B) in P7 NDUFS2-deficient mice. In addition, the level of *Ndufs2* mRNA in CBs from hGFAP-NDUFS2 mice was similar to its value in CBs from heterozygous (*Ndufs2^flox^*^/^*^-^*) mice, thereby suggesting that hGFAP-Cre dependent recombination had not taken place (Figure 5C). To confirm this observation, we performed a lineage-tracing experiment using hGFAP-Cre mice bred with a *Rosa26* knock-in tdTomato reporter strain, and analyzed the progeny produced in the CB 1 week after birth. Notably, numerous tdTomato positive cells, probably representing GFAP^+^ type II cells (Pardal et al., 2007; Macías et al., 2014) were detected in CBs from P7 mice (Figure 5D). However, consistent with the data shown above, at P7 we did not observe TH^+^ cells that were also tdTomato positive (Figure 5D,E). Interestingly, analysis of cell fate at longer ages (P21) revealed some TH^+^ cells also marked with tdTomato (Figure 5F), suggesting a glial (GFAP^+^) origin. The absence of hGFAP-Cre mediated *Ndufs2* deletion in neuron-like glomus cells at P7 (age at which hGFAP-NDUFS2 mice died) prevented us from studying the impact of MCI dysfunction on postnatal CB maturation. In any instance, our data suggest that, during the first week after birth newborn CB glomus cells either do not significantly originate from GFAP^+^ precursors or they differentiate from cells without an active hGFAP promoter.

### NDUFS2 Deficiency Reduces ATP Production and Impairs Proliferation in Postnatal Neural Stem/Progenitor Cells

**FIGURE 6:**
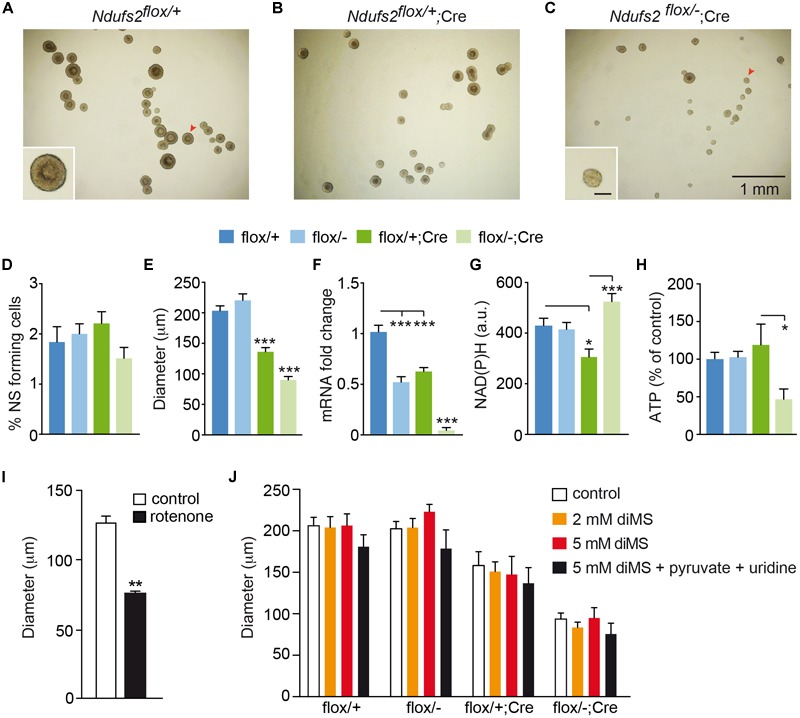
NDUFS2 deficiency reduces ATP production and impairs proliferation of postnatal neural stem/progenitor cells. **(A–C)** Bright-field images of SVZ-derived neurospheres from P7 mice (7-day culture) of the indicated genotype. The insets show higher magnifications of representative neurospheres (red arrowheads). Inset scale bar: 100 μm. **(D–H)** Quantitative analyses of SVZ-derived neurospheres (7-day culture) from P7 wild-type (flox/+) and *Ndufs2* and hGFAP-Cre mutant (flox/–; flox/+Cre; flox/-Cre) mice. Data are presented as mean ± SEM. ^∗^*p* < 0.05, ^∗∗∗^*p* < 0.001 (One Way ANOVA and Tukey’s *post hoc* test). **(D)** SVZ-neurosphere forming efficiency (*n* = 4–7 mice/group). **(E)** Core diameter (*n* = 4–8 mice/group). **(F)** *Ndufs2* mRNA levels (*n* = 4–6 mice/group). **(G)** Quantification of basal NAD(P)H autofluorescence levels (a.u., arbitrary units) (*n* = 28–51 cells/group). **(H)** Relative ATP content (*n* = 5 mice/group). **(I)** SVZ-neurosphere core diameter in 7-day cultures from P30 wild type mice (*n* = 6 mice). Neurospheres were cultured in control conditions or in the presence of 10 nM rotenone (*n* = 4 independent experiments). Data are presented as mean ± SEM, ^∗∗^*p* < 0.01 (unpaired Student’s *t*-test analysis). **(J)** SVZ-neurosphere core diameter in 7-day cultures from P7 mice. Neurospheres were cultured in control conditions (control) or in the presence of different metabolites. Pyruvate and uridine were added at 2 mM and 0.1 mg/mL, respectively. Data are presented as mean ± SEM (*n* = 3–12 mice/condition). Data were analyzed by Two Way ANOVA and Tukey’s *post hoc* test. diMS: dimethyl succinate.

Although central neurogenesis and gliogenesis mostly occur during embryonic life, they also take place after birth (Frisén, 2016). Adult mammalian NSCs reside mainly in two regions, the SVZ of the lateral ventricles and the subgranular zone of the dentate gyrus in the hippocampus (Ming and Song, 2005; Kriegstein and Álvarez-Buylla, 2009; Dimou and Götz, 2014). NSCs from the SVZ can give rise to intermediate progenitors, which differentiate into neuroblasts that migrate along the rostral migratory stream into the olfactory bulb, where they differentiate into interneurons (Lim and Álvarez-Buylla, 2014). As the hGFAP promoter is active in rodent NSCs from the SVZ (Pastrana et al., 2009; Beckervordersandforth et al., 2010), we investigated the effect of MCI dysfunction on these cells using our hGFAP-NDUFS2 model. To evaluate the survival and proliferation of NSCs, we performed *in vitro* neurosphere assays of isolated SVZ cells from hGFAP-NDUFS2 mice and from control littermates (Figure 6A–C). Given that neurospheres are formed by NSCs and intermediate progenitor cells (Pastrana et al., 2011; Gil-Perotín et al., 2013), hereafter we use the term neural stem/progenitor cell (“NSPC”) to include these two cell populations (Taverna et al., 2014). The number of SVZ-derived neurospheres was slightly lower in MCI mutant mice compared with the control animals but the differences were not statistically significant (Figure 6D). In contrast, a clear decrease in neurosphere diameter in hGFAP-NDUFS2 mice was observed (Figure 6A–C,E), indicating that NSPCs proliferation was seriously compromised by MCI dysfunction. In these experiments down-regulation of *Ndufs2* mRNA in the SVZ neurospheres (resulting from *Ndufs2* ablation) was confirmed by quantitative PCR analysis (Figure 6F). Moreover, using single cell microfluorimetry we showed increased basal levels of NAD(P)H autofluorescence in hGFAP-NDUFS2 neurospheres (Figure 6G), which is a hallmark of MCI-deficient cells (Fernández-Agüera et al., 2015; Arias-Mayenco et al., 2018). The similar number of SVZ-derived neurospheres in the hGFAP-NDUFS2 mice in comparison with controls suggests that survival of NSPCs was not severely affected, whereas the decrease of neurospheres diameter in the hGFAP-NDUFS2 mice indicates that proliferation of central NSPC population was inhibited by MCI dysfunction. Total ATP levels, measured in SVZ-derived neurospheres, were only decreased in homozygous MCI-deficient cells (Figure 6H). Surprisingly, a significant impairment in proliferation was also detected in SVZ neurospheres from Cre-induced heterozygous mice (*Ndufs2^flox^*^/+^; hGFAP-Cre). This finding, not studied in detail, could be explained by a possible deleterious side effect of hGFAP-Cre expression itself or by a combined toxicity resulting from Cre expression in the *Ndufs2* heterozygous background (see section “Discussion”). Nevertheless, in a similar hGFAP-Cre background, *Ndufs2*-null NSPCs showed a more pronounced (and significant) defect in proliferation than the *Ndufs2^flox^*^/+^; hGFAP-Cre mice indicating the relevance of a functional MCI in that process. Moreover, pharmacological inhibition of MCI with rotenone in wild type mice also impaired NSPCs proliferation, thus confirming the specific effect of MCI genetic ablation in the hGFAP-NDUFS2 model (Figure 6I). Together, these data suggest that NSPCs proliferation requires a functional MCI and electron transport chain (ETC) as well as ATP synthesis.

Interestingly, both SCG neurons and neuron-like CB glomus cells appear to be resistant to *Ndufs2* deletion and are able to maintain normal ATP levels in the absence of a functional MCI (Fernández-Agüera et al., 2015). To explain these findings it has been proposed that induction of glycolysis, uptake of exogenous pyruvate, and a highly efficient MCII succinate dehydrogenase activity could support ETC and mitochondrial function to permit cell survival in a decreased MCI activity scenario (Arias-Mayenco et al., 2018). In addition to the production of ATP, an essential function of mitochondrial metabolism in proliferating cells is to support the production of aspartate required for nucleotide synthesis (Birsoy et al., 2015; Sullivan et al., 2015). Uridine is also required for pyrimidine synthesis when the ETC is not functional (Grégoire et al., 1984). Exogenous pyruvate (converted to lactate in the cells) increases the NAD^+^/NADH ratio and thus provides electron acceptors for Krebs’s cycle dehydrogenases required for succinate metabolism and complex II-mediated ETC (see Arias-Mayenco et al., 2018). Therefore, pyruvate supplementation can revert the anti-proliferative effect of MCI dysfunction (Birsoy et al., 2015; Sullivan et al., 2015). Based on these observations, we tested in our SVZ neurosphere preparation several protocols to overcome MCI dysfunction in *Ndufs2* knockout NSPCs. Incubation with different doses of dimethyl succinate (a permeable MCII substrate) did not cause any change in SVZ-neurosphere diameter (Figure 6J). Addition of pyruvate and uridine to the NCM also failed to stimulate MCI-deficient NSPCs proliferation (Supplementary Figure S3). Moreover, the combined addition of pyruvate, uridine and dimethyl succinate did not revert the proliferative defect of *Ndufs2* null NSPCs (Figure 6J). Therefore, our data show that NSPCs from the SVZ require a functional MCI-mediated ETC and OxPhos activity to support *in vitro* proliferation.

### NSPCs Differentiation Into Neurons and Oligodendrocytes Is Impaired in NDUFS2 Deficient Mice

**FIGURE 7:**
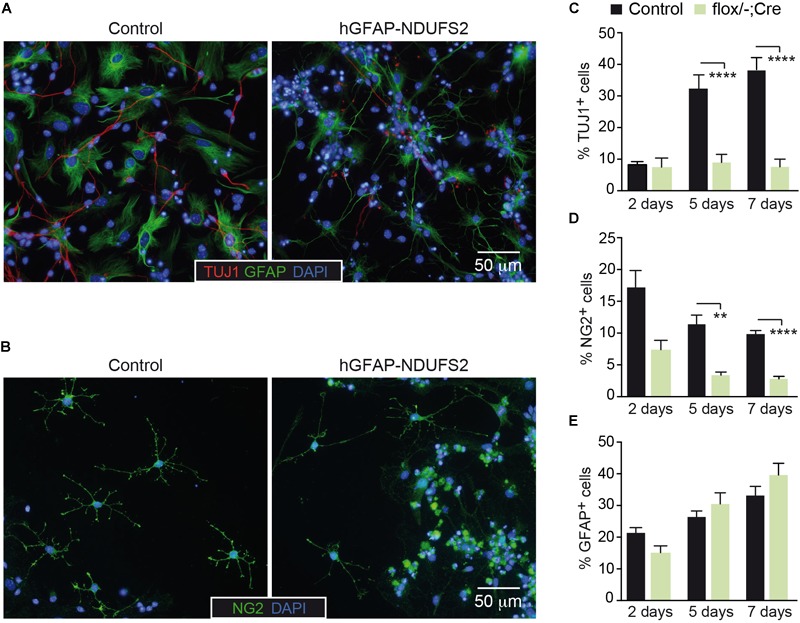
NSPCs differentiation into neurons and oligodendrocytes is impaired in NDUFS2 deficient mice. **(A,B)** Immunofluorescence detection of TUJ1 and GFAP **(A)** or NG2 **(B)** in SVZ-derived neurosphere adherent cultures (5 days after mitogens retrieval) from P7 *Ndufs2^flox/+^* control (left panels) and hGFAP-NDUFS2 (right panels) mice. Nuclei were counterstained with DAPI. **(C–E)** Percentage of TUJ1^+^ **(C)**, NG2^+^ **(D)**, and GFAP^+^ **(E)** cells (relative to total number of blinded counted cells) in SVZ-derived neurospheres cultured under adherent conditions (and without mitogens) for 2, 5, and 7 days. The results from the hGFAP-NDUFS2 mice littermates (flox/+; flox/–; flox/+Cre) were pooled together and assigned to a control group (control) as no differences were detected among them (*n* = 4 independent experiments; *n* = 13 mice for control group and *n* = 6 hGFAP-NDUFS2 mice). Data are presented as mean ± SEM. ^∗∗^*p* < 0.01, ^∗∗∗∗^*p* < 0.0001 (multiple *t*-test analysis, statistical significance determined using the Holm-Šidák method).

To further investigate the impact of MCI dysfunction on neurogenesis and gliogenesis, SVZ-derived neurospheres were subjected to neural differentiation-promoting conditions. SVZ NSCs have the potential to differentiate *in vitro* into astrocytes, oligodendrocytes and neurons (Reynolds and Weiss, 1992; Weiss et al., 1996). Immunofluorescence analysis revealed the presence of all three neural lineages in cultures from hGFAP-NDUFS2 and control mice (Figure 7A,B), indicating that neurogenic and gliogenic potential was preserved in MCI-deficient NSPCs. Nonetheless, we found considerable differences in viability among the three cell lineages from MCI knockout neurospheres compared with the progeny of homozygous and heterozygous control littermates. There was a marked decrease in the percentage of newly generated neurons from hGFAP-NDUFS2 mice at the endpoint of the differentiation assay, as indicated by the labeling with the early postmitotic neuronal marker TUJ1 (Figure 7A,C). Time-course analysis showed that the percentage of TUJ1^+^ neurons was similar in control and *Ndufs2* knockout neurospheres 2 days after induction of differentiation (Figure 7C, 2 days). However, whereas the number of TUJ1^+^ cells progressively increased with time in control neurospheres, this was not observed in cultures from hGFAP-NDUFS2 mice (Figure 7C, see progression from 2 to 7 days). These data indicate that neuronal differentiation and/or survival of differentiated neurons is markedly compromised in cultures from MCI-deficient SVZ–NSCs, thereby supporting the essential role of OxPhos in neurogenesis. The yield of oligodendrocyte progenitor cells was also significantly lower in MCI deficient cultures, suggesting that oligodendrocyte differentiation requires a normal OxPhos function (Figure 7D). In sharp contrast with the effects on the generation of neurons and oligodendrocytes, *in vitro* astrocyte differentiation and survival did not appear to be severely affected by MCI dysfunction (Figure 7E). This observation is consistent with the view that astrocytes can show a highly glycolytic profile (Bolaños et al., 2010; Bélanger et al., 2011; d’Anglemont de Tassigny et al., 2015). A proportion of GFAP^+^ cells with an elongated appearance could be detected in cultures of SVZ cells from control and hGFAP-NDUFS2 mice. However, cells with an elongated shape were more abundant in the knockout differentiation assays (Figure 7A). This morphology could be indicative of an intermediate state of differentiation and resembled the GFAP^+^ cells with long processes detected in the hGFAP-NDUFS2 brains (Figure 4A–D). Thus, our results provide further evidence for the distinct metabolic requirements of the different neural lineages and their precursors.

## Discussion

Mitochondria are organelles needed for cellular energy production and intermediate metabolism. They also participate in the synthesis of precursors required for essential cell functions (e.g., nucleotides or heme, among others), signaling processes, free radical production and apoptosis. The relevance of mitochondrial metabolism and dynamics in developmental and adult neurogenesis has gained substantial attention in recent years. In addition to their essential role in neuronal differentiation and survival (Homem et al., 2014; Khacho et al., 2016; Zheng et al., 2016), there is increasing evidence supporting the importance of mitochondria in other neural processes such as NSCs self-renewal capacity, fate decision, and proliferation (Beckervordersandforth et al., 2017; Khacho et al., 2017, 2019). These recent observations are mostly based on genetic models that induce severe mitochondrial defects. Conditional loss of the mitochondrial protein AIF (apoptosis-inducing factor) has been used to analyze the effects of mitochondrial dysfunction in developmental neurogenesis. AIF deficiency causes mitochondrial fragmentation and loss of OxPhos function (Germain et al., 2013; Khacho et al., 2017) and in the context of NSPCs, it has been shown to interfere with self-renewal capacity, proliferation and differentiation (Khacho et al., 2017). Disruption of mitochondrial function has also been assessed by ablation of the mitochondrial transcription factor A (*Tfam*) in adult hippocampal NSCs, causing defects in proliferation and viability of intermediate progenitors (Beckervordersandforth et al., 2017). TFAM plays an essential role in mitochondrial biogenesis (Larsson et al., 1998) and its depletion induces profound changes in mitochondria morphology and function (Beckervordersandforth et al., 2017). Hence, the specific contribution of mitochondrial ETC and OxPhos in neurogenesis remains poorly understood. Genetic deletion of *Ndufs2* abolishes MCI enzymatic activity, leaving a potentially functional MCII-MCIV pathway (Fernández-Agüera et al., 2015). In addition, deregulation of MCI has been associated with neurodevelopmental and neurodegenerative disorders (Lin and Beal, 2006; Carelli and Chan, 2014; Giachin et al., 2016) and thus appears as an appealing candidate to analyze its specific contribution to neurogenesis.

Here, we show that MCI ablation mediated by hGFAP-Cre/loxP recombination in NSCs induces early perinatal death and causes major defects in the CNS, compromising especially the postnatal development of dorsal cortex, corpus callosum, hippocampus and cerebellum. Neonatal neurogenesis and gliogenesis are deeply impaired in hGFAP-NDUFS2 mice. MCI dysfunction provoked a decrease in proliferation and induction of apoptosis in the altered brain areas. These results uncover the involvement of MCI in the survival of proliferating neural progenitors and highlight the relevance of mitochondrial metabolism in the viability of the differentiating neural progeny. On the other hand, the absence of phenotype in ventral telencephalon can be explained by the fact that hGFAP promoter is active in the cortex at around E12.5 whereas hGFAP driven expression in the ganglionic eminences does not occur until E14.5 and thus fails to target neurons in the ventral telencephalon (Anthony and Heintz, 2008). Interestingly, the fact that the brain of newborn hGFAP-NDUFS2 mice is similar to that of control mice suggests that NSCs are not severely affected by MCI dysfunction during embryonic development. In accord with this observation, *in vitro* analysis of postnatal NSPCs from the SVZ indicated that survival of these cells was not compromised, as the number of MCI-deficient neurospheres was the same as the controls. These data support the view that NSCs are predominantly glycolytic and can survive MCI dysfunction.

In agreement with the *in vivo* observations in the hGFAP-NDUFS2 P7 brains, *in vitro* proliferation of postnatal SVZ–NSPCs is highly dependent on MCI function and proliferating NSPCs from *Ndufs2* knockout mice show deficient ATP production. In line with these results, the study of Beckervordersandforth et al. (2017) using *Tfam* conditional knockout cultures from the adult hippocampal neurogenic lineage, has shown that the fast proliferating progenitor cell population also depends on mitochondrial integrity to divide. Aberrant NSPCs *in vitro* proliferation was also observed in a mouse model of MCII genetic inactivation (Díaz-Castro et al., 2015). Altogether, these results challenge the view that proliferative neural intermediate progenitors are mostly glycolytic (Agathocleous et al., 2012; Candelario et al., 2013; Knobloch and Jessberger, 2017) and set a new scenario to consider regarding NSPCs metabolism, placing the so-called “metabolic switch” from glycolysis to OxPhos at an earlier stage. Interestingly, neither MCII stimulation (by succinate addition) nor pyruvate supplementation could revert the low proliferative capacity of *Ndufs2* knockout NSPCs, indicating that proliferating postnatal neural progenitors specifically require a functional MCI-dependent ETC and OxPhos. Also, the SVZ *in vitro* assays revealed that MCI dysfunction affects differentiation and survival of the three neural lineages (neurons, astrocytes, and oligodendrocytes) in a different manner, being the astrocytes the population more resistant to MCI deficit.

Our experiments with NSPCs cultures revealed the striking observation that proliferation was affected in the hGFAP-Cre-induced heterozygous mice (*Ndufs2^flox/+^* hGFAP-Cre), although to a lesser extent than the hGFAP-NDUFS2 mice (*Ndufs2^flox/-^* hGFAP-Cre). This phenomenon could be explained by a possible deleterious side effect of hGFAP-Cre expression itself or to a combined toxicity resulting from Cre expression in the *Ndufs2* heterozygous background. Neurosphere assays using hGFAP-Cre mice in a wild type background revealed that the proliferation defect could be caused solely by hGFAP-Cre expression (data not shown). This observation, far from being an extraordinary episode, brings back the issue that Cre-lox system is not absent from side effects (Heffner et al., 2012; Harno et al., 2013; Song and Palmiter, 2018). Remarkably, a specific “toxic” effect of Cre expression in the brain has been shown (Forni et al., 2006; Qiu et al., 2011; Harno et al., 2013). These observations highlight the importance of including Cre-expressing controls for an adequate interpretation of data. In these sense, all experiments performed with the hGFAP-NDUFS2 mouse model were analyzed separately for every genotype and the only unexpected phenotype for the *Ndufs2^flox/+^* hGFAP-Cre littermate mice was the effect on perinatal NSPCs *in vitro* proliferation. Notably, ATP content in neurospheres from those mice was unaffected, indicating that the proliferative defect was not caused by a deficit in energy production. On the other hand, the marked reduction of ATP levels in hGFAP-NDUFS2 NSPCs and a significantly stronger defect in proliferation compared with the *Ndufs2^flox/+^* hGFAP-Cre cells support the specific impact of MCI dysfunction on the proliferative capacity of NSPCs regardless of the Cre-expression phenotype. In line with these observations, pharmacological inhibition of MCI also led to impaired proliferation.

Considering the profound alterations that MCI dysfunction caused in the brain of hGFAP-NDUFS2 mice, we were also interested in assessing whether neurogenesis and/or neuronal viability in peripheral nervous system could also be affected by MCI genetic ablation. As previous studies suggested that hGFAP-Cre mediated recombination could take place in postnatal CB GFAP^+^ stem cells (Díaz-Castro et al., 2015), we tried to investigate the impact of MCI specific deletion in hGFAP-NDUFS2 mice on perinatal production of CB neuron-like cells. However, *Ndufs2* expression analysis and lineage-tracing experiments suggest that during the first week after birth, newborn CB glomus cells do not originate from GFAP^+^ precursors. Alternatively, it could also be feasible that perinatal hyperplasic maturation of the CB would indeed depend on GFAP^+^ stem cells but a low efficiency of hGFAP-driven Cre expression in those cell types would not elicit recombination. Remarkably, lineage-tracing experiments at later stages (3 weeks after birth) support the hypothesis that postnatal maturation of mouse CB depends (at least partially) on glia-like GFAP^+^ cells.

In summary, our data reveal that MCI function is essential for mammalian perinatal central neurogenesis and also affects gliogenesis. Although NSCs appear to be resistant to MCI dysfunction, neural progenitors require a functional MCI-dependent ETC and OxPhos to produce ATP and to proliferate. The differentiation of NSCs to a neuronal or glial lineage and subsequent survival of newly generated cells are processes differentially affected by MCI deficiency. Thus, this study contributes to unravel the importance of mitochondria in NSC function, addressing relevant aspects of the specific impact of MCI genetic ablation on neurodevelopment and SVZ postnatal neurogenesis.

## Data Availability

All datasets generated for this study are included in the manuscript and/or the Supplementary Files.

## Ethics Statement

The animals were maintained before, during and after the experiments according to EUROPEAN DIRECTIVE 2010/63/EU regarding the use of experimental animals and other scientific purposes (ROYAL DECREE 53/2013, February 8). All procedures were reviewed and approved by the Ethics Committee of Animal Experimentation (CEEA/CEI) of Hospital Virgen del Rocío/Institute of Biomedicine of Seville (reference number 22-09-15-332).

## Author Contributions

DC-R, HS-S, and AM-C performed the experiments. DC-R, JL-B, and AM-C designed the study and prepared a draft of the manuscript. JL-B and AM-C coordinated the project and wrote the manuscript.

## Conflict of Interest Statement

The authors declare that the research was conducted in the absence of any commercial or financial relationships that could be construed as a potential conflict of interest.

## Abbreviations

αKB: alpha-ketobutyrate
AM: adrenal medulla
ANOVA: analysis of variance
CB: carotid body
CNS: central nervous system
DAPI: 4′,6′-diamidino-2-phenylindole
hGFAP: human glial fibrillary acidic protein
EBSS: Earle’s balanced salt solution
ETC: electron transport chain
MCI: mitochondrial complex I
NCM: neurosphere culture medium
NSCs: neural stem cells
NSPC: neural stem/progenitor cell
OxPhos: oxidative phosphorylation
PBS: phosphate-buffered saline
PFA: paraformaldehyde
RGCs: radial glial cells
SCG: superior cervical ganglion
SEM: standard error of the mean
SVZ: subventricular zone
TH: tyrosine hydroxylase
