# Supplementary material for: Mitochondrial Complex I Function Is Essential for Neural Stem/Progenitor Cells Proliferation and Differentiation
Source: Front Neurosci. 2019 Jun 26;13:664. doi: 10.3389/fnins.2019.00664 (PMC6607990; doi:10.3389/fnins.2019.00664)
Supplement: Supplementary file 1 [file Data_Sheet_1.PDF]

Supplementary Figure S1

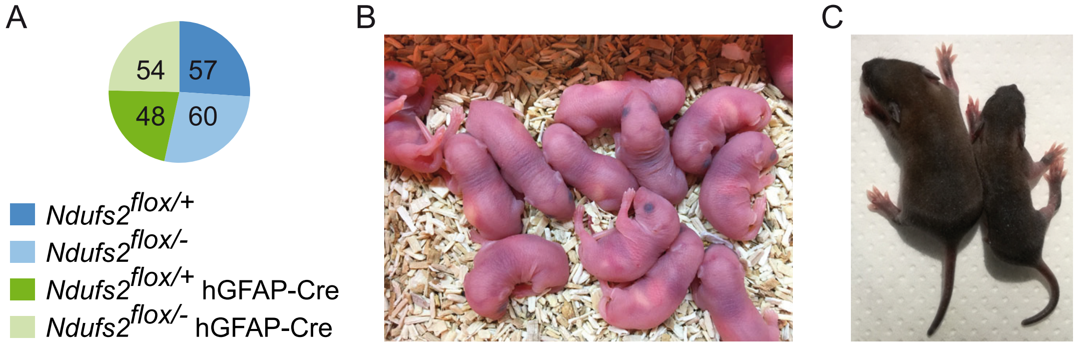

Figure S1. (A) Distribution of the offspring generated by crossing *Ndufs2*$^{flox/flox}$ with *Ndufs2*$^{+/-}$; hGFAP-Cre mice. The number of analyzed mice is indicated for each genotype. Mutant mice were born at the expected Mendelian ratio (Chi-squared test). **(B)** Photograph of a P0 litter that included three hGFAP-NDUFS2 mice. **(C)** Photograph of a P7 *Ndufs2*$^{flox/+}$ control mouse (left) and a hGFAP-NDUFS2 littermate mouse (right).

Supplementary Figure S2

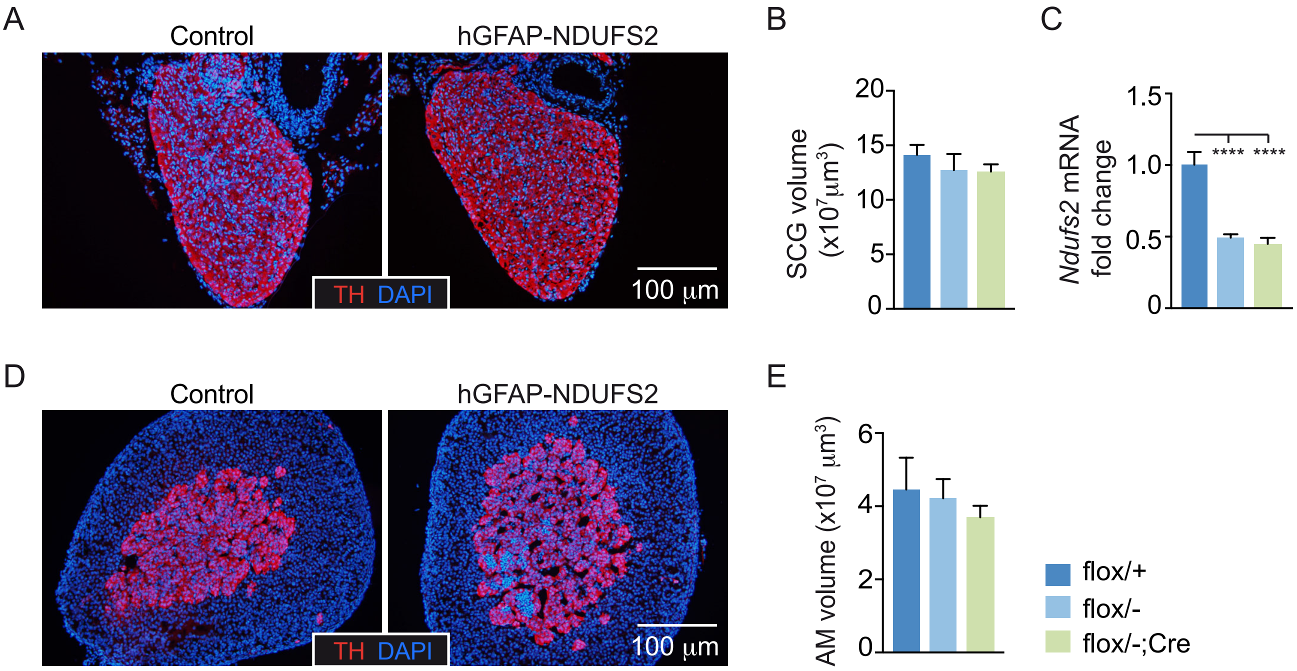

Figure S2. Effect of NDUFS2-deficiency on peripheral neural tissues in P7 mice. (A) Immunofluorescence detection of TH (red) in SCG sections in *Ndufs2*$^{flox/+}$ control (left) and hGFAP-NDUFS2 (right) mice. Nuclei were counterstained with DAPI (blue). **(B)** SCG volume (n= 4-9 mice/group). **(C)** *Ndufs2* mRNA levels in SCG (n=3 experiments from pooled samples). **(D)** Immunofluorescence detection of TH (red) in AM sections in *Ndufs2*$^{flox/+}$ control (left) and hGFAP-NDUFS2 (right) mice. Nuclei were counterstained with DAPI (blue). **(E)** AM volume (n= 3-10 mice/group). Data are presented as mean ± SEM. ****p<0.0001 (One Way ANOVA and Tukey's *post hoc* test).

Supplementary Figure S3

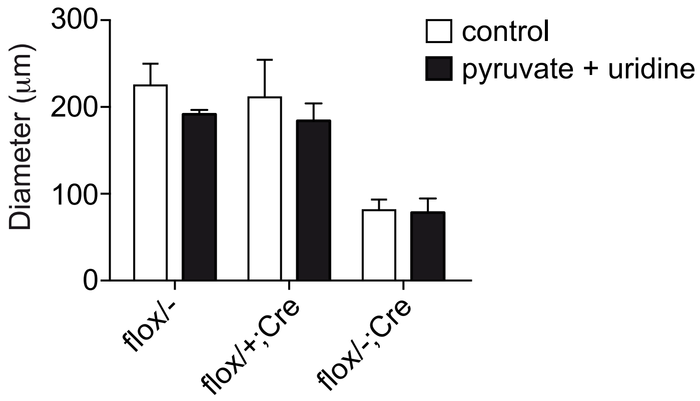

Figure S3. Pyruvate and uridine addition does not stimulate MCI-deficient NSPCs proliferation. SVZ-neurosphere core diameter in 7-day cultures from P7 *Ndufs2* and hGFAP-Cre mutant (flox/-; flox/+Cre; flox/-Cre) mice. Neurospheres were cultured in control conditions or in the presence of 2 mM sodium pyruvate and 0.1 mg/mL uridine. Data are presented as mean ± SEM (n=2 mice/condition).
